# The Differences in Antibiotic Decision-making Between Acute Surgical and Acute Medical Teams: An Ethnographic Study of Culture and Team Dynamics

**DOI:** 10.1093/cid/ciy844

**Published:** 2018-11-15

**Authors:** E Charani, R Ahmad, T M Rawson, E Castro-Sanchèz, C Tarrant, A H Holmes

**Affiliations:** 1 Health Protection Research Unit in Healthcare-Associated Infections and Antimicrobial Resistance, National Institute for Health Research, Imperial College London; 2 Department of Health Sciences, University of Leicester, United Kingdom

**Keywords:** antimicrobial decision-making, culture, team dynamics

## Abstract

**Background:**

Cultural and social determinants influence antibiotic decision-making in hospitals. We investigated and compared cultural determinants of antibiotic decision-making in acute medical and surgical specialties.

**Methods:**

An ethnographic observational study of antibiotic decision-making in acute medical and surgical teams at a London teaching hospital was conducted (August 2015–May 2017). Data collection included 500 hours of direct observations, and face-to-face interviews with 23 key informants. A grounded theory approach, aided by Nvivo 11 software, analyzed the emerging themes. An iterative and recursive process of analysis ensured saturation of the themes. The multiple modes of enquiry enabled cross-validation and triangulation of the findings.

**Results:**

In medicine, accepted norms of the decision-making process are characterized as collectivist (input from pharmacists, infectious disease, and medical microbiology teams), rationalized, and policy-informed, with emphasis on de-escalation of therapy. The gaps in antibiotic decision-making in acute medicine occur chiefly in the transition between the emergency department and inpatient teams, where ownership of the antibiotic prescription is lost. In surgery, team priorities are split between 3 settings: operating room, outpatient clinic, and ward. Senior surgeons are often absent from the ward, leaving junior staff to make complex medical decisions. This results in defensive antibiotic decision-making, leading to prolonged and inappropriate antibiotic use.

**Conclusions:**

In medicine, the legacy of infection diagnosis made in the emergency department determines antibiotic decision-making. In surgery, antibiotic decision-making is perceived as a nonsurgical intervention that can be delegated to junior staff or other specialties. Different, bespoke approaches to optimize antibiotic prescribing are therefore needed to address these specific challenges.

## Teaser

Culture and team dynamics within specialties influence antibiotic decision-making. A collectivist culture in medicine supports rationalized and policy-driven decision-making. In surgery, antibiotic decision-making is delegated to juniors or other specialties. Addressing these specific challenges is critical to optimizing antibiotic prescribing.

**(See the Editorial Commentary by Szymczak on pages 21–3.)**

Across hospitals, healthcare professionals from a range of specialties diagnose and treat community- and hospital-acquired infections. Antimicrobial stewardship programs (ASPs) are implemented in hospitals to optimize antibiotic use [1–3]. These programs are not, however, contextually designed or implemented with end users of different specialties in mind. In healthcare, culture influences the shape and outcome of interventions and impacts on patient outcomes [4, 5]. Culture has been defined as “the shared knowledge people use to interpret, experience, and generate behavior” [6] as members of a group. The key components of culture are norms, values, tools, and rituals that people of a specific group adopt, that identify them as belonging to that group [6, 7]. To optimize patient care, hospital-based specialties constantly diversify and evolve in response to scientific and technological breakthroughs and developing technical expertise. This diversification creates microcultures within the macro healthcare culture [8, 9]. The microcultures within specialties have the potential to shape intervention outcomes.

**Figure 1.**
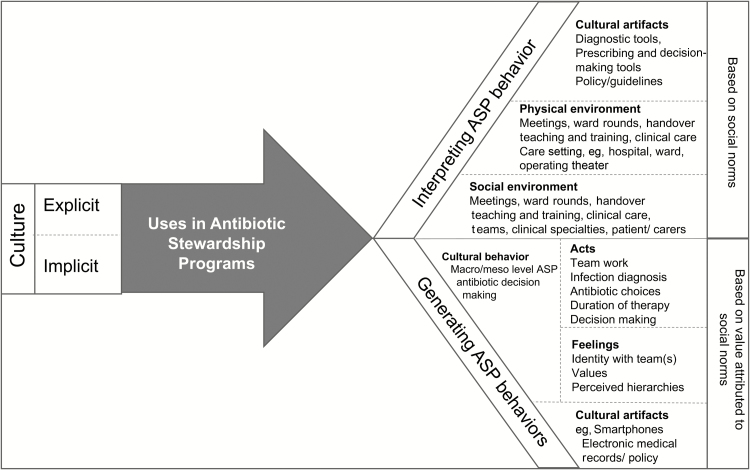
The model of culture used in this study to study antibiotic decision-making [6]. Abbreviation: ASP, antibiotic stewardship program.

ASP studies emphasize quantitative methodologies, to answer “what works,” and “by how much” with an emergent body of research investigating “why” interventions do or do not work [10]. Studies have explored the contextual determinants of antibiotic prescribing decision-making, identifying the influence of hierarchies and etiquette [11], and the complexities of rising to expectations set in policies and guidelines [12]. Such insights are critical for the development of targeted and sustainable ASP. Qualitative research methods provide in-depth understanding of the subject being studied leading to development of new theories [6, 13]. Ethnographic studies in hospitals provide a rich understanding of the contextual complexities that influence outcomes [14–16]. Qualitative research methods have investigated education and training of doctors [17, 18], and effective team communication [19, 20] on ward rounds (WRs). These studies report on the complexity of WRs. In surgery, studies have used WR simulation to test interventions including efforts to reduce disruptions, and checklists to improve patient outcomes [21–23]. We conducted a mixed-methods study of antibiotic decision-making with a focus on WRs in medicine and surgery. The study included ethnographic research into how culture and team dynamics within specialties affect antibiotic decision-making. We drew on Spradley’s [6] definition of culture to define and describe the cultural norms and values that help generate behaviors (Figure 1). In this article, we describe the qualitative findings, focusing on the influence of culture and team dynamics on antibiotic decision-making. These insights are important in the development of contextually fit ASPs.

## METHODS

Ethical approval for this study was granted by the UK National Research Ethics Committee (application number 176038).

### Sampling and Data Collection

#### Ward Sample

The study was conducted in a central London teaching hospital with 1300 beds and an existing ASP [24]. Wards were conveniently sampled to represent a high percentage of elective admissions, and nonelective admissions; wards were identified through consultation with medical directors at the hospital. Clinical leaders on the identified medical and surgical wards identified were emailed the study information sheet and invited to participate in the study. All healthcare professionals who attended the WRs or worked on the study wards were eligible to participate in the study. The lead consultant surgeons/physicians who agreed to participate in the study were asked to provide a list of the names of the staff who participated on the WRs. An information leaflet was sent to all participants; to reduce bias and Hawthorne effect [25], the leaflet stated that the study investigated clinical decision-making. Informed consent was obtained before the observations and interviews. The interviews took place following the observations to ensure that participants could be questioned about their antibiotic decision-making without affecting their behaviors during the observations.

### Data Collection Methods

#### Ethnographic Observations

Ethnography is the study of people within the context in which they exist, live, and work [6, 13]. The ethnographic study design included nonparticipant direct observations, interviews, and documentary analysis (detailed notes on the type of data collected are provided as Supplementary Data). One researcher, E. C., conducted the observations on the wards and face-to-face interviews with key informants. Detailed, descriptive notes of observations were collected. Separate reflective notes were kept detailing the observer’s perceptions and interpretations of what was recorded. Handover sheets, multidisciplinary team meeting notes, and the policy and guidelines on antibiotic prescribing were collected to provide contextual knowledge of the processes. These different methods supported cross-validation and triangulation of the findings.

#### Face-to-Face Interviews

Healthcare professionals participating in the observations were invited to participate in a follow up face-to-face interview. The interviews were semistructured with an interview guide, developed through review of literature and drawing upon previous work of the research team [11]. The interviews were recorded using an audio recorder, transcribed verbatim, and anonymized.

### Data Analysis

The data were analyzed using classic grounded theory approach [26], using mainly inductive methods of inquiry. Grounded theory relies on simultaneous data collection and analysis, in an iterative manner that enables theory construction and does not rely on existing frameworks for analysis. The analysis aimed to explore categories and relationships within the data collected. During focused coding, a constant comparative method was used for the analysis of the emerging categories and themes [27], aided by Nvivo 11 software. The data from observations, documentary analysis, and interviews were open coded to identify key categories, which were developed into themes. The analysis was conducted using an iterative and recursive process of moving between the coded data, data collection in the field, and the higher-level themes, until the themes and the relationship between the themes reached saturation (ie, no new themes or interrelationships between them were identified).

## RESULTS

**Figure 2.**
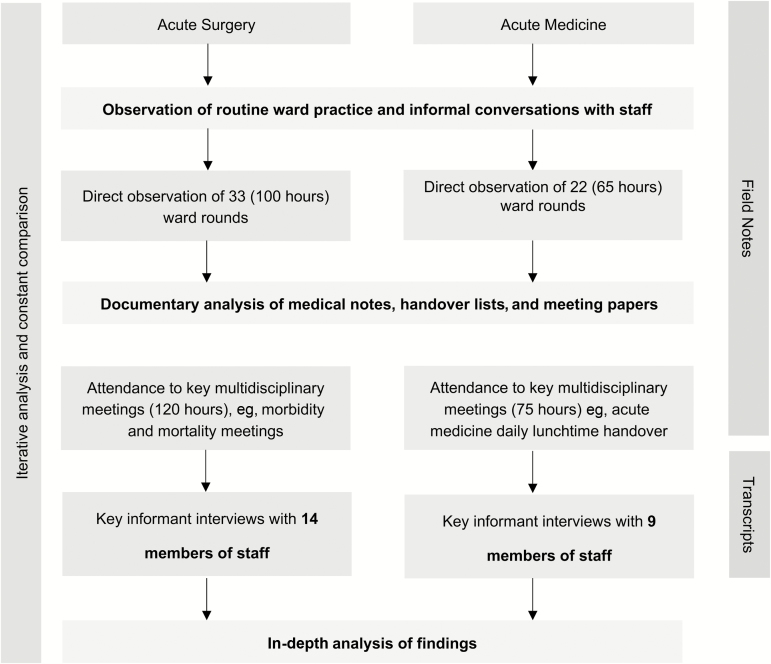
The data gathering and analysis process.

The results presented below are derived from the analysis of field notes, documents, and the interview data. The field notes and quotes in this article are representative of the key emerging themes. Between August 2015 and May 2017, 30 surgical and 22 medical WRs (Figure 2) were observed. More than 100 hours of observation of routine healthcare worker practices were also collected from the wards. The acute surgical team was comprised of 6 surgeons, and the acute medical team of 14 consultants. Twenty-three healthcare professionals (14 in surgery, 9 in medicine) including surgeons, medical consultants, trainee doctors, nurses, and pharmacists were interviewed (Supplementary Data).

### Differences in Team Dynamics and Characteristics of the Teams

**Figure 3.**
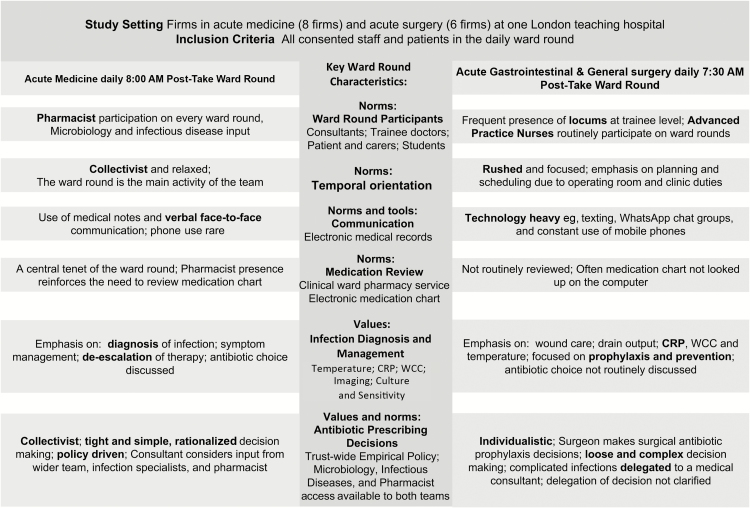
The key team dynamics and characteristics of the ward rounds (derived from field notes). Abbreviations: CRP, C-reactive protein; WCC, white cell count.

In surgery, individualism is clear in the influence of consultant surgeons on the team [7, 28]. The surgical team is vertical in structure, with the surgeons leading decision-making and with less room or opportunity for team input, particularly during WRs (Figure 3). In medicine, a more collectivist culture prevails, whereby each team member is encouraged to participate. The medical WR included a dedicated pharmacist (Table 1, quote [Q] 1). The presence of the pharmacist reinforces the necessity to review patient medications; the pharmacist will often question whether the antibiotic therapy could be de-escalated from intravenous to oral therapy or switched to a narrower-spectrum agent according to the local policy (Table 1, Q1).

**Table 1.** Key Emerging Themes

Theme	Quote
Ward round characteristics	Q1: “The consultant asks the night team ‘what have you done?’ referring to any tests and examinations the team may have done overnight. He asks what the patient white cell count and C-reactive protein is. ‘What about antibiotics? What have you given him?’ The junior doctor answers that the emergency department [ED] team started the patient on piperacillin-tazobactam, and together with the consultant they reason that is an appropriate choice considering the patient is admitted from rehab. They then look at the laboratory results. The consultant sees the patient and decides to continue the antibiotics and also prescribe the patient furosemide. The pharmacist checks with the consultant if they are treating the patient with antibiotics for hospital acquired pneumonia because he’s been admitted from rehab. The consultant confirms this to be correct.” —*Field notes, Medicine*
	Q2: “If you’re a surgeon you don’t want to be on the ward, you’d rather be in clinic than on the ward. And it’s where you start off learning about patients really.” —*Interview, Surgical Intern*
	Q3: “Leaving the ward the junior doctor continues: ‘What I hate about surgery is that the ward rounds are done in such a rush, we never get to delve into the patient history, in medicine there is more delving into the detail…. he has been in our care for 3 days and we didn’t know he has bronchiectasis.’ The surgical ward rounds are very intervention based. They are very rapid, the team, especially the seniors are under pressure to do round fast to go back to theatre.” *—Field notes, Surgery*
	Q4: “If there was a war we’d all die, if we were special ops we’d get shot, it’s just not, they’re not thinking big plan format.” —*Interview, Surgeon B*
	Q5: “You said it quite rightly, it’s disseminated, it’s like a puzzle. Different people hold different aspects of it, particularly it’s not just the medical staff, nursing staff and therapy staff and social workers.” —*Interview, Medical Intern A*
	Q6: “There’s microbiology ward rounds … I think the [infectious disease consultant] has revolutionized our perception of treating infections, so I think a liaison service of someone of his personality who is enthusiastic, engaging, kind, and considerate. I think that’s the real change that’s required. What we need is a cultural change, and that is done through human interactions, not through facts and information. So, giving people facts isn’t going to change their perception, but heightened cultural awareness is, and so I think we need to engage people at a human level to make that change.” —*Interview, Medical Intern B*
	Q7: “The pharmacists here on the ward round are really important. They, because the ethos driving us is so difficult and so confusing that, without them, I think there’d be a lot more errors and they will always point out what antibiotic they’re on and that.” —*Interview, Medical Intern C*
Uncertainty and a fear of blame	Q8: “If my patient gets a wound infection, for example, my case will be discussed at a Morbidity and Mortality meeting…it affects my data … and my outcome data will be on a website, so I’m going to practice pretty defensive medicine, absolutely.” —*Interview, Surgeon F*
	Q9: “It’s the culture, it’s easy, it’s too easy to say put them on Tazocin because I don’t want my operation screwed up.” —*Interview, Surgeon B*
	Q10: “The intern goes to speak to the surgeon leading the ward round. I ask the junior doctor about the antibiotics that have been prescribed for the patients on the ward round. Specifically, about the piperacillin-tazobactam prescribed for the 2 patients. The junior doctor replies: ‘It is the surgeon’s choice for the patients… sometimes we treat with antibiotics when we can’t find evidence for infection. We are not as strict as pharmacy when it comes to antibiotics … we have to keep patients safe. There have been examples in the past when patient was not given antibiotics and developed an infection.” —*Field notes, Surgery*
	Q11: We operate in gray areas, it’s rare that there’s 100% specific certainty. [In surgery] you’re either cutting someone open or you’re not, you’re either removing something or you’re not and I think when you add in medical decision-making in to surgery, that doesn’t really work, I think they have to know and so if you’re thinking, there might be an infection and you’ve already made a decision to literally cut someone open, uncertainty, they just don’t like that, and fair enough I think you’ve got to be really confident to cut a human being in bits, I think that’s not something I would fancy doing and I think it just is going to be a different mindset. It’s a different situation obviously as well, there’s the consequences I guess of getting it wrong are perhaps different as well both in terms of real outcomes but also maybe psychological that if you’ve cut someone open and then they get a horrible infection and they die, you look like an idiot. Whereas if one of my patients gets a horrible infection and dies, it’s not really my fault, whether it was avoidable or not, it just doesn’t feel like that.” —*Interview, Consultant E*
	Q12: “The major challenge is this, is that, there is a fundamental difference in medicine and surgery, which is if someone comes in with a pneumonia, so you try and treat it, but that person with pneumonia dies. Well you tried and that’s OK. If someone comes in to hospital for an elective operation, and they die from sepsis or infection, that death was preventable and it’s your fault. And therefore, surgeons practice an incredibly defensive brand of medicine, and if there is even a small chance that me giving a dose of prophylactic antibiotics or keeping my patients on 10 days instead of 7, and it means that my patient’s outcome will be better, and my outcome data will be better, because I get judged, then I’m going to give that patient antibiotics. I’m going to do it, and so I think what you see is a lot of surgeons prescribe defensively, and they don’t really care what the evidence is, and they don’t really care what the problems [of] antibiotic resistance are. So I think that’s the major hurdle you’ve got to get over. And that’s a real challenge, because it’s not just providing an evidence base, you’re changing the entire culture.” —*Interview, Surgeon F*
	Q13: “There’s a lot of gray areas and if, in medicine, you didn’t take any risks, or any perceived risks, so things that seem like real risks to someone who’s inexperienced, someone who’s being doing the job for 20 years sees those risks as different and says, I’ve seen this 100 times …. if we stop the antibiotics here and see what happens for the next 24 hours, 48 hours … this might not be an infection…. I think there’s the part that’s there to make sure I haven’t missed anything serious, and there’s the part that’s there to actually de-escalate everything and go, well actually I think that we’re overdoing it now. And if we take a step back here there’ll be no harm done. Because it’s taking a risk on behalf of my consultant. When I’m a consultant, which I will be in a few months, it’s a slightly different game … it’s up to me and where my own risk compass lies in my barometer of these decisions. But it’s hard to second guess someone else and what they’d like.” **—***Interview, Medical Intern C*
	Q14: “I think escalation is clearer, like I said, the consultant’s got 2 roles. One is to see what I’ve missed, the other is to de-escalate everyone being overzealous. And so the de-escalation’s something that’s definitely, I think, in the more senior camp. Whereas the escalation, I think the junior doctors are more likely to escalate than de-escalate. And I think that goes with the whole risk profile and nervousness.” —*Interview, Medical Intern C*
	Q15: “I think it is the same with the junior doctors as well that they are more scared of not giving them because of the risk of what could happen vs the risk of giving them because, I think it’s to do with risk because it’s more risky not to give it.” —*Interview, Pharmacist A, Medicine*
Legacy of infection diagnosis in ED determines antibiotic decision-making in medicine	Q16: “I do sometimes feel that people are inappropriately started on antibiotics and you think what, what’s the likely gain here? But it’s very difficult to do nothing, well it’s not very difficult, but you, you feel that you’re going to be criticized if you don’t do anything.” —*Interview, Consultant C*
	Q17: “You can be a little tied up, because as I say if somebody’s been on a certain treatment for a few days and they’re getting better, you slightly worry that if you downgrade or change then you may end up halting the improvement, if you go I don’t think I would have given this person Tazocin, but they’ve had it for 3 days and they’ve gone from being moribund to sitting up chatting away, well it’s quite difficult to then go well actually I would have given them 3 days of trimethoprim and that would have been it.” —*Interview, Consultant E*
	Q18: “The junior doctor replies that no blood cultures had been sent. The consultant replies that ‘For all the people with sepsis we’ve got to make people realize they have to take blood culture before starting antibiotics.’ The intern comments on the difficulty of getting the diagnosis of sepsis right: ‘Anyone with a temperature is defined as having sepsis.’ Intern 2: ‘I have given up with ED trying to define what sepsis is with them, now I just roll over.” —*Field notes, Medicine*
	Q19: “We’re often making decisions so early, that you don’t even notice whether they’re [results] back or not, it doesn’t matter, you know you’re not going to have any relevant information so you just get on and make a decision anyway, and it’s only when you bother to check back, but we’re so focused on the start of the admission, the front door and all that kind of thing, we’re getting a culture result 4 days later, if it pops up something interesting that’s great…and then you go, did we even send a culture, no, oh well bit late now…. But you don’t even know what’s gone. The only time you can guarantee getting an MSU [midstream urine] is if you’ve asked for urinary electrolytes and you can absolutely guarantee that the electrolytes weren’t sent, but MSU were sent….” —*Interview, Consultant E*
	Q20: “It certainly is a different approach than with other medications. The main difference being that you’re picking the antibiotic in a time when you probably don’t know the diagnosis for sure. And it’s easy enough if you’ve got an x-ray that shows pneumonia. But quite often … it is a bit less of a definite diagnosis.” —*Interview, Medical Intern C*
	Q21: “So I think there’s 2 big things and both are areas of uncertainty. The first one is, is this an infection at all? Because obviously there’s people who come in with clear criteria for sepsis and a focus and you say this is a sepsis syndrome and we know there’s a valid response, we know there’s a focus, we’ve got a rough idea what it’s likely to be, and we’ve got a protocol for treating and that’s fine ... [second uncertainty] is a lot of the time it’s not as clear cut as that, people come in nonspecifically unwell, and older people they may not develop a full obvious systemic inflammatory response, they might have a slightly raised inflammatory markers, they might have a bit of a temperature but it’s not anything specific and there’s no … symptoms, so first of all is there infection at all? And then secondly if there is, where is it likely to be? And that determines whether we give antibiotics at all or we watch and wait, because if we are giving antibiotics, which ones we should use based on what we think the likelihood of the underlying focus is. But, I think the first one is probably the more difficult one because the acute medical model, the focus on your first day in hospital, your first few hours in hospital is very much on somehow, despite all this uncertainty, we can make a decision immediately and set the course of your treatment.” —*Interview, Consultant E*
	Q22: “I think there’s probably far too much acceptance that once somebody’s made that decision, and it’s not always a consultant who’s made that decision, that actually everyone might as well carry on. I think it’s partly the training here. I think it’s that, if somebody makes it through ED, makes into medical admission, it’s quite hard to do nothing. It’s hard to justify admitting them if you’re not doing very much. And I think in a younger person that is even more difficult.” –*Interview, Consultant A*
	Q23: “I think there are improvements to be made in ED with what they start on, I’m always amazed at how much co-amoxiclav I see prescribed by ED. I have sympathies, they’re under pressure, they do more empirical treatment than we do. We’re seeing someone that maybe a few more hours down the line where they’re a bit more stable and there might be a clearer picture of where infection lies. Even so, co-amoxiclav is not in any guidance in the sense as far as I’m aware. But it still gets dished out … and it generally gets dished out in ED. I think almost as often as not, maybe 50% of the time it gets continued because it’s been started. So, I think there’s improvements to be made at where antibiotics is started.” —*Interview, Pharmacist A*
	Q24: “I think it’s more fear than trust. I think the person who gets to know the patient best is the, is probably the junior doctor who clerks in a patient. And everyone from that person onwards is making an impression based upon information that they have to trust from someone else. So, I can see how there’s reluctance to change something that someone else has started, who you probably think knows the patient, and has got to grips with the situation better than you have. Plus, the longer that goes on, a day, 2 days, 3 days, you’re like, well, it’s not the best antibiotic but they’ve already had 2 days’ worth of it, you’ll continue that for the 5-day course.. I think that where to intervene is at the beginning and as early on as possible before things get down the line.” —*Interview, Medical Intern C*

How time is viewed and managed is an important attribute of each specialty and determines the collective behavior of the teams. The sense of time in the medical team is less structured or pressurized. In contrast, the surgical team focus primarily on the “now,” requiring the team to plan and schedule, and have a sense of the time taken up by the WR, and the need to end it and move on to other tasks (Table 1, Q2 and 3). The surgical team has 3 main commitments including the operating room, clinics, and the ward. The surgeons spend much of their time in the clinics or operating room (Table 1, Q2). This leads to teams being dispersed, and working in a disjointed manner, with little opportunity for forward planning (Table 1, Q4). As a result, surgical teams rely much more on their mobile telephones for team communication, including decisions about patient care that occur via messaging apps such as WhatsApp (Figure 3). The medical teams are more likely to have face-to-face meetings, and it is rare for mobile phones to be used on the WR. One of the contributing factors to the communication challenges in the surgical team is the lack of multidisciplinary input into patient care on the WR. As in the surgical observations, information is diffuse among the team. However, the medical team are more in tune with the need to have access to multidisciplinary staff during the WR and the review of patients (Table 1, Q5–7). The physical presence of the infectious disease consultant removes a communication barrier, with the team often requesting and receiving ad hoc advice for individual patients (Table 1, Q6).

In surgery, the diagnosis of infection is reliant on infection markers, including C-reactive protein, white cell count, and temperature. The decision-making is focused more on prevention and prophylaxis than on treatment of infections. In medicine, though infection markers are an important part of the process, the team members try to rationalize their decisions, making efforts to align them with local policy and readily involve other healthcare professionals.

### Uncertainty and a Fear of Blame

The need and expectation to intervene in surgery means that antibiotics are readily initiated for surgical patients. This process is rationalized by the surgeons as being an extension of their roles as “interventionists” [16]. In the absence of concrete evidence of infection, what drives decision-making is not the risk of failure, but the risk of blame (Table 1, Q8 and Q9). A focus on starting, but not on reviewing or stopping, treatment can lead to unnecessary and prolonged courses of antibiotics (Table 1, Q10). The awareness of the culture of blame and responsibility that was evident in the surgical team is reflected in the medical team’s perception of how antibiotic decision-making occurs in surgery. The impetus to “do something” is greater in surgery, as “patients are not allowed to die” (Table 1, Q11 and Q12).

In medicine, junior members were concerned about managing risks in the context of making decisions on behalf of senior leaders. The junior doctors are more likely to start and escalate antibiotic therapy but are reliant on consultants deciding whether therapy needs to be de-escalated (Table 1, Q13 and Q14). The reluctance of the trainee doctors to de-escalate is tied to this acute awareness that decisions will be viewed as ultimately being made on behalf of the consultant who is responsible for the care of the patient, highlighting ambiguity about ownership for antibiotic decision-making (Table 1, Q15).

### Legacy of Infection Diagnosis in the Emergency Department Determines Antibiotic Decision-making in Medicine

In medicine, the critical decisions about whether a patient needs to be prescribed antibiotics occur in the first 24 hours of admission. There are multiple teams involved in the care of the patient, presenting challenges for assuming ownership of antibiotic decision-making. The participants identified problems arising from pressure to act and respond rapidly in the emergency department (ED) (Table 1, Q16 and Q17). Some of this urgency to initiate antibiotics revolves around the ED clinicians having a heightened awareness of the need to rapidly diagnose and treat sepsis (Table 1, Q18). The decision-making often takes place without samples taken for microbiological investigations (Table 1, Q19 and Q20). There is also a perception that often antibiotics are initiated upon admission to buy time for the clinicians and to act as an exercise in uncertainty avoidance (Table 1, Q21). This decision to initiate therapy where there is a suspicion of infection becomes difficult to evaluate and review on subsequent WRs, particularly if patient symptoms are improving. There is a reluctance to change or override the decisions made in ED (Table 1, Q22–Q24). This reluctance to assume responsibility for the antibiotic decision-making made in the ED is not replicated for other classes of medication. Anticoagulants, analgesics, and blood pressure medications are amended during the WR and adjusted based on the patient symptoms. Cardiology issues are routinely apportioned to the cardiologist intern based on the admissions ward (field notes). It is only for the antibiotic therapy that the teams report and exhibit a hesitation to alter the prescribing decisions of their colleagues (Table 1, Q17).

## DISCUSSION

The patient care pathway in hospitals includes a multitude of medical and surgical specialties, pharmacists, nurses, and allied healthcare professionals, who may prioritize different policies, agendas, and interim goals. Different specialties have their own language, behaviors, social norms, and values. The cultural differences between specialties and healthcare professionals: (1) shape the shared knowledge within and across specialities in patient pathways, and (2) result in variation in care, thus impacting patient outcomes. Antibiotic decision-making is driven by different determinants in medicine vs surgery. There are different drivers of overuse that arise from cultural and contextual differences across these specialties. An understanding of these key differences is essential to the development of contextually fit and sustainable interventions to optimize antibiotic use.

The interplay of collectivist vs individualistic approaches, together with fear of negative outcomes in surgery, leads to less tolerance to uncertainty. In medicine, uncertainty arises from the transition of care between ED and inpatient teams. The medical teams take a more collectivist approach than surgical teams. Though subject to communication flaws, the medical team addresses them by including other healthcare professionals, such as the pharmacist and infectious diseases consultant, in routine, daily decision-making. The presence of the pharmacist on the WR and the infectious diseases consultant on the ward means that they can have direct discussions with the consultants and decisions can be made there and then. The cultural norm of collectivism and the interdisciplinary approach to decision-making contribute to efforts to optimize antibiotic use. The gaps in care around antibiotic decision-making in medicine appear to be in the 24 hours after admission, when the patient moves from ED to medical wards. This transition of care between different teams is a challenge to antibiotic optimization. The Surviving Sepsis Campaigns have driven the culture of being “infection aware” and initiating antibiotics for patients upon admission [29], but can have the unintended consequence of driving inappropriate antibiotic use in the ED. The medical team in this study reported frustrations at antibiotics being initiated in the ED that are then not reviewed or de-escalated. Though it can be accepted that patients may be started on empirical antibiotics with little evidence of infection in the first hours of admission, it is not acceptable for patients to remain on antibiotics unnecessarily once the medical teams have had time to clinically review and reassess patients. The pharmacists have in this study been identified as healthcare professionals who can oversee and promote rationalized antibiotic decision-making, if they are part of the WR. If supported by the wider team, they may be able to address the gaps in ownership in antibiotic prescribing in the transition between the ED and inpatient teams for medical patients. The transition from the ED to surgical inpatients was less problematic as the surgical team assumed responsibility for patients identified as needing surgical attention in the ED. There is no transition of patients between teams, and the surgical team remains in charge of the patient for the duration of the admission episode.

The surgical team however, has its own unique challenges. Having the senior surgeons in theater and the junior doctors chasing medical jobs means that the skill mix of the team on the ward is sometimes imbalanced. Difficult decisions such as de-escalation of antibiotic therapy are often deferred. This may result in prolonged and unnecessary courses of antibiotics for surgical patients. The surgeons prioritize surgical and patient outcomes, and their fear of negative patient outcomes overrides the fear of the unintended consequences of inappropriate antibiotic use. Time is limited in the surgical team due to the demands from the clinics, operating room, and wards. The different professions do get involved in the surgical patient pathway, but they tend to work in silos, with few communication opportunities between them. Multidisciplinary teamwork is not easily practiced in such a context, and time constraints are part of the problem. The surgeons’ time on the ward is extremely limited, so they must prioritize their commitments, preferring to only communicate with their own team and expecting the junior members of the team to coordinate the multidisciplinary tasks and actions that are identified. Therefore, it may not be expedient to suggest replicating the acute medical model of including a pharmacist on surgical WRs. Equally it may be unrealistic to expect surgeons to include ASPs in their daily review of patients. We identified inconsistencies in the approach to antibiotic decision-making in surgery, often with the key identified variables not discussed as part of the WR. Some of this is due to the practicalities of the WRs in surgery and can be overcome through simple solutions (Table 2). The surgeons, though not fully engaged with the antibiotic decisions made for their patients, remain the leaders in their specialty and engaging with them in ASP will be tantamount to engaging with their entire team. They have the power to influence the behaviors of their entire team. When constructing ASPs targeting surgical teams, the language and metrics must reflect the priorities of surgical teams, who are focused on outcomes. Using length of hospital stay and surgical outcomes may be more convincing measures than antibiotic consumption rates. It is also important to recognize the different work patterns and shifts of surgeons, and to accommodate these differences in the effort to include them in ASPs. For example, the surgical handover is an opportunity for engaging with the surgical team. The first 30 minutes to 1 hour is dedicated to detailed discussion of patients, including those admitted via the ED. Pharmacist and/or infectious diseases specialists can participate in the handover to discuss antibiotic- and infection-related issues for the patients. The surgical team uses messaging groups and texting as a preferred means of communication. Developing a specialty-level ASP-specific group on a messaging platform may get the attention of the team more effectively than the pharmacist trying to page the junior team, who must ratify any decisions verbally or via messages with the senior team. Becoming more engaged and involved with the surgical teams in the management and prevention of infection will also provide opportunities for the ASP teams to understand the priorities and difficulties in the surgical pathway. This will help them develop more context-driven interventions that are more likely to be adopted by the surgeons. Furthermore, it may result in a more interdisciplinary ASP in surgery.

**Table 2.** Opportunities in Antibiotic Management in Surgery and Medicine

	Observed Practice	Opportunities
Surgery	• Senior surgeons are not actively engaged in antibiotic decision-making • Lack of coordinated communication about antibiotic management of surgical patients • Diffuse responsibilities for antibiotic management	• The surgeons are the leaders in their specialty; engaging with a surgeon is tantamount to engaging with their entire team • Colleagues with expertise in antibiotics (microbiology/infectious disease and pharmacy) should engage and communicate with surgeons in a consistent and sustainable way, this includes accommodating the different working patterns of surgeons, eg, in this study an ideal point of daily intervention and engagement is the 7.30 AM WR, where the team spends the first 30 minutes to discuss and present every patient to the lead surgeon • It is critical to engage with the surgical teams on the communication platforms most frequently used by them; this may be via phone, text messaging, etc • Define a dedicated clinical role for antibiotic stewardship within the surgical team, this can be context specific whether it is a pharmacist or a nurse or surgical trainees who have responsibility for ensuring appropriate antibiotic management for patients in their team
Medicine	• The lack of communication in transition of care between ED and medical teams leading to prolonged inappropriate antibiotics initiated in ED	• Target ASP interventions on the first 48 hours after admission to rationalized antibiotics started in ED. Having a clinical pharmacist as part of medical WRs will assist with ensuring that antibiotic decisions are not overlooked and therapy is appropriate

Abbreviations: ASP, antibiotic stewardship program; ED, emergency department; WR, ward round.

This is a single-center study limited to acute medical and surgical teams in one hospital in England. While the findings may be broadly applicable to other settings, we are replicating the study in centers in India and South Africa investigating antibiotic decision-making across surgical pathways. The study methodology has also been replicated in a study in Australia.

## CONCLUSIONS

The observed variation in the social norms, values, and behaviors in medicine and surgery defines the approach to antibiotic decision-making. The medical team adopts a more policy-driven, interdisciplinary approach that includes pharmacist and infectious diseases input. The transition of care between the ED and inpatient care is a critical point for optimizing antibiotic prescription decisions in medicine. The surgical teams perceive themselves to be highly skilled interventionists, and see ASPs as having a low priority. Medical and surgical specialties represent different tribes, with different cultures and challenges. To optimize antibiotic use across these specialties, it is imperative that interventions are designed with these unique challenges in mind.

## Supplementary Data

Supplementary materials are available at *Clinical Infectious Diseases* online. Consisting of data provided by the authors to benefit the reader, the posted materials are not copyedited and are the sole responsibility of the authors, so questions or comments should be addressed to the corresponding author.

ciy844_suppl_Supplementary_Material_Data_Collection

ciy844_suppl_Supplementary_Material_Participants

ciy844_suppl_Supplementary_Material

## Notes

***Financial support.*** This work was funded by Economic and Social Science Research Council as part of the Antimicrobial Cross Council initiative [ES/P008313/1] supported by the seven UK research councils, and the Global Challenges Research Fund and the National Institute for Health Research, UK Department of Health [HPRU-2012-10047] in partnership with Public Health England.

***Potential conflicts of interest.*** E. C.-S. has received consulting fees from Pfizer. A. H. has received royalties from Medical Services, MSD Hoddesdon, and Imperial College London. All other authors report no potential conflicts. All authors have submitted the ICMJE Form for Disclosure of Potential Conflicts of Interest. Conflicts that the editors consider relevant to the content of the manuscript have been disclosed.
