# Supplementary material for: The Differences in Antibiotic Decision-making Between Acute Surgical and Acute Medical Teams: An Ethnographic Study of Culture and Team Dynamics
Source: Clin Infect Dis. 2018 Nov 15;69(1):12–20. doi: 10.1093/cid/ciy844 (PMC6579961; doi:10.1093/cid/ciy844)
Supplement: ciy844_suppl_Supplementary_Material_Data_Collection [file ciy844_suppl_supplementary_material_data_collection.doc]

**Supplementary Material**

**The differences in antibiotic decision-making between acute surgical and acute medical teams – an ethnographic study of culture and team dynamics**

Authors: Charani E, Ahmad R, Rawson TM, Castro-Sanchèz E, Tarrant C, Holmes A

Corresponding author:

Esmita Charani

NIHR Health Protection Research Unit

Healthcare Associated Infections and Antimicrobial Resistance

Hammersmith Campus

W12 ONN

Email: [e.charani@imperial.ac.uk](mailto:e.charani@imperial.ac.uk)

| **Episode of Observation** | **Ward practice** | **Ward Rounds** | **Multidisciplinary Meetings** | **Shadowing individuals** |
| --- | --- | --- | --- | --- |
| **Data Collected** | Duration  The time of day  A general description of the ward layout  Number of bay and side beds  A description of the activities taking place  A description of the people working on the ward  The patterns of activity | Duration  People in attendance  Who lead the ward round  The number of wards visited  The number of patients visited  What was discussed – who lead the discussions, who contributed  What tools were used e.g. electronic prescribing, smartphones  What tasks were identified  Who was responsible for carrying out tasks  What interactions there were with patients  What interactions there were with other healthcare professionals  What each member of the team did during the ward round i.e. what they contributed or if not contributing what they were doing  Any emotions expressed or felt  Observer contribution, if any, to the activities e.g. pulling curtains, getting gloves for consultant  Any disruptions to the activity | Duration  Meeting type e.g. Morbidity & Mortality meetings  Who attended  What was discussed  Who lead the discussion  Who contributed to the discussion  What, if any, data was used or presented  Who presented the data  Any emotions expressed or felt | Duration  Type of activity e.g. nurse medication administration  Any dialogue between observer and participant  Any disruptions to the activity  The interactions of the healthcare professional with patients and other members of staff  Places visited  Tools used e.g. guidelines, electronic systems  Observer contribution, if any, to the events taking place e.g. helping the pharmacist/ doctors with the electronic prescribing trolley |

Table Describing the type of data collected from the ethnographic observations
