# Supplementary material for: The Differences in Antibiotic Decision-making Between Acute Surgical and Acute Medical Teams: An Ethnographic Study of Culture and Team Dynamics
Source: Clin Infect Dis. 2018 Nov 15;69(1):12–20. doi: 10.1093/cid/ciy844 (PMC6579961; doi:10.1093/cid/ciy844)
Supplement: ciy844_suppl_Supplementary_Material_Participants [file ciy844_suppl_supplementary_material_participants.doc]

**Supplementary Material**

**The differences in antibiotic decision-making between acute surgical and acute medical teams – an ethnographic study of culture and team dynamics**

Authors: Charani E, Ahmad R, Rawson TM, Castro-Sanchèz E, Tarrant C, Holmes A

Corresponding author:

Esmita Charani

NIHR Health Protection Research Unit

Healthcare Associated Infections and Antimicrobial Resistance

Hammersmith Campus

W12 ONN

Email: [e.charani@imperial.ac.uk](mailto:e.charani@imperial.ac.uk)

| Specialty | Profession | Years in Practice | Country of post-graduate training | Gender |
| --- | --- | --- | --- | --- |
| Surgery | Surgeon | 2 | UK | M |
|  | Nurse | 1 | UK | F |
|  | Attending Physician | 2 Months in UK | Egypt | M |
|  | Intern | 2 Months in UK | China | M |
|  | Intern | 4 Months | UK | F |
|  | Attending Physician | 2 | UK | F |
|  | Surgeon | 11 | UK | M |
|  | Surgeon | 2 | UK | M |
|  | Pharmacist | 15 | UK | F |
|  | Surgeon | 1 | UK | M |
|  | Attending Physician | 3 | UK | F |
|  | Consultant | 3 | UK | M |
|  | Nurse | 15 | UK | F |
|  | Surgeon | 15 | UK | M |
| Medicine | Consultant | 25 | UK | M |
|  | Attending Physician | 1 | UK | M |
|  | Attending Physician | 3 | UK | M |
|  | Consultant | 5 | UK | M |
|  | Attending Physician | 6 | UK | M |
|  | Pharmacist | 4 | UK | F |
|  | Consultant | 24 | UK | M |
|  | Consultant | 6 | UK | M |
|  | Pharmacist | 4 | UK | F |
