# Supplementary material for: The Differences in Antibiotic Decision-making Between Acute Surgical and Acute Medical Teams: An Ethnographic Study of Culture and Team Dynamics
Source: Clin Infect Dis. 2018 Nov 15;69(1):12–20. doi: 10.1093/cid/ciy844 (PMC6579961; doi:10.1093/cid/ciy844)
Supplement: ciy844_suppl_Supplementary_Material [file ciy844_suppl_supplementary_material.docx]

**Supplementary Material**

**The differences in antibiotic decision-making between acute surgical and acute medical teams – an ethnographic study of culture and team dynamics**

Authors: Charani E, Ahmad R, Rawson TM, Castro-Sanchèz E, Tarrant C, Holmes A

Corresponding author:

Esmita Charani

NIHR Health Protection Research Unit

Healthcare Associated Infections and Antimicrobial Resistance

Hammersmith Campus

W12 ONN

Email: [e.charani@imperial.ac.uk](mailto:e.charani@imperial.ac.uk)

**SEMI-STRUCTURED QUESTIONNAIRE FORM STAFF**

(These are template pointer questions, which will be supplemented by further questions that will arise as a result of the observational study)

1. Do you think the ward round is the right place **to discuss management of infections**?
   1. Why, do you think so? (ask if response to above is positive or negative)
2. Do you think **antimicrobial decisions are made at ward round**?
3. Can you describe to me **what role you think you have in decision making** around antimicrobial prescribing/administration/monitoring
   - 1. **on the ward round?**
     2. **in general**
4. Do you **feel involved in the decision making** process at the ward round?
5. Who in your opinion is **the person who makes the key decisions** on antimicrobial prescribing
   1. **In the ward round?**
   2. **In general?**
6. Do you have **confidence in your seniors/juniors ability** to prescribe antibiotics?
7. Is there **clear leadership** when it comes to making decisions on antimicrobial prescribing?
8. **Who in the team** would you say has the most influence on your own prescribing decisions?
9. **Should patients be involved** in antimicrobial decision making in hospitals?
10. Can you describe a typical consultant/surgeon attitude to antimicrobial prescribing for their patients?
11. Can you describe a typical registrar attitude to antimicrobial prescribing for their patients?
12. Can you describe a typical junior doctor attitude to antimicrobial prescribing for their patients?
13. Can you describe a typical nurse attitude to antimicrobial prescribing for their patients?
14. Can you describe a typical pharmacist attitude to antimicrobial prescribing for their patients?
